# *O*-glycosylated pro-B-type natriuretic peptide is cleaved intracellularly by furin in ventricular and atrial myocytes: importance of the distance between the *O*-glycosylation and cleavage sites

**DOI:** 10.1186/2050-6511-14-S1-P50

**Published:** 2013-08-29

**Authors:** Toshio Nishikimi, Yasuaki Nakagawa, Naoto Minamino, Masashi Ikeda, Toshihiko Ishimitsu, Chinatsu Yamada, Kazuhiro Nakao, Takeya Minami, Yoshihiro Kuwabara, Hideyuki Kinoshita, Koichiro Kuwahara, Kenji Kangawa, Kazuwa Nakao

**Affiliations:** 1 Department of Medicine and Clinical Science, Kyoto Univ. Graduate School of Medicine, Japan; 2 Department of Hypertension and Cardiorenal Medicine, Dokkyo Medical University, Japan; 3 Department of Laboratory Medicine, Dokkyo Medical University, Japan; 4 Research Institute National Cardiovascular Research Center, Japan; 5 Department of Cardiology, Fujii Hospital, Japan

## Background

Our objective was to investigate the molecular mechanism underlying the processing of proBNP, levels of which are increased in heart failure [1,2].

## Methods

Rat neonatal atrial and ventricular myocytes were cultured separately. We examined: (1) the molecular forms of secreted and intracellular BNP in atrial and ventricular myocytes; (2) levels of corin and furin mRNA in atrial and ventricular myocytes and the effect their knockdown on proBNP processing; (3) molecular forms of BNP in plasma from rats and humans with and without heart failure; (4) the structure of proBNP in humans and rats; (5) the impact of the distance between the glycosylation and cleavage sites in wild-type and mutant human proBNP expressed in rat myocytes transfected with lentiviral vectors.

## Results

**Figure 1:**
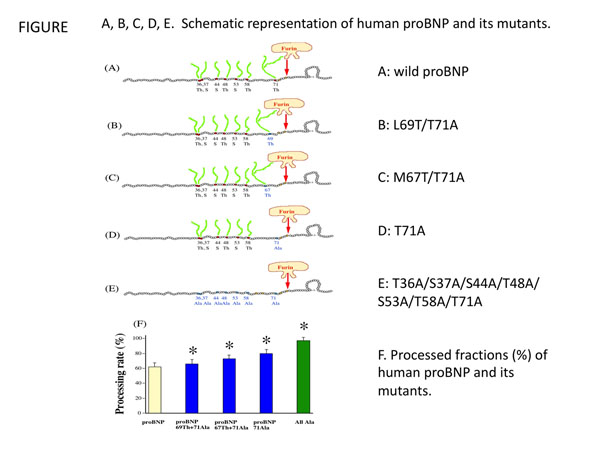


BNP was the major molecular form secreted by atrial (75±9%) and ventricular (85±9%) myocytes, and was the major intracellular form in atrial myocytes (60±5%). ProBNP was the major intracellular form in ventricular myocytes (58±4%). The relative levels of furin mRNA correlated with those of BNP in atrial and ventricular myocytes, and transfection of furin siRNA reduced proBNP processing in both atrial and ventricular myocytes. BNP was the major molecular form in rat plasma (90±10%), whereas proBNP was the major form in human plasma (72±8%). The relative fraction of human BNP in rat myocytes expressing human proBNP was 60±5%, but increasing the distance between the glycosylation and cleavage sites through mutation, increased the processed fraction correspondingly (Figure 1).

## Conclusion

These results suggest that proBNP is processed into BNP and N-terminal proBNP intracellularly, most likely by furin in rat. The level of proBNP processing is lower in humans than rats, most likely due to the smaller distance between the *O*-glycosylation and cleavage sites in humans.
